# Inflammatory Joint Diseases

**DOI:** 10.1155/2015/973510

**Published:** 2015-05-21

**Authors:** Guixiu Shi, Julian L. Ambrus, Shuang Ye, Long Shen

**Affiliations:** ^1^Department of Rheumatology and Clinical Immunology, The First Hospital of Xiamen University, Xiamen 361003, China; ^2^Buffalo General Medical Center, State University of New York at Buffalo School of Medicine, Buffalo, NY 14203, USA; ^3^Department of Rheumatology, Renji Hospital, Shanghai Jiao Tong University School of Medicine, Shanghai 200001, China; ^4^Division of Allergy, Immunology and Rheumatology, Department of Medicine, State University of New York at Buffalo School of Medicine and Biomedical Sciences, Buffalo, NY 14203, USA

Arthritis is a common clinical manifestation of rheumatic diseases. It can be a clinical feature in many rheumatic diseases, including rheumatoid arthritis, spondyloarthritis, crystal-induced arthritis, systemic lupus erythematosus, and Sjogren's syndrome. Joint inflammation and damage may result in disability and morbidity. Understanding the pathogenesis of inflammatory joint diseases remains a complex problem, although the level of understanding has progressed considerably in recent years. Knowledge of the pathogenesis, diagnosis, and treatment of inflammatory joint arthritis will lead to significant clinical benefit.

Based on this background, we assembled this special issue for presenting recent advances and a better understanding of inflammatory joint diseases, on aspects of pathogenesis, diagnosis, and treatment of inflammatory joint arthritis, including rheumatoid arthritis, spondyloarthritis, and osteoarthritis.

In this special issue, V. Romão compared effectiveness of tocilizumab and TNF*α* inhibitors in rheumatoid arthritis patients, showing that tocilizumab was associated with greater likelihood of achieving DAS28, CDAI, and SDAI remission/LDA and EULAR good response. I. Arstikyte analyzed the influence of immunogenicity on the efficacy of long-term treatment with TNF*α* blockers in rheumatoid arthritis and spondyloarthritis patients. H. Cho studied the effects of cyclooxygenase-2 (COX2) inhibitor and steroids on matrix metalloproteinases (MMPs) and prostaglandin E2 (PGE2) production in osteoarthritis patients, demonstrating that celecoxib and steroids exert similar effects on MMP-1 and PGE2 production in vitro and that celecoxib may demonstrate favorable effects on anabolic metabolism in vivo. X. Cen studied the association between serum 25-hydroxyvitamin D level and rheumatoid arthritis. G. Yin demonstrated that Pim-2/mTORC1 pathway shapes inflammatory capacity in rheumatoid arthritis synovial cells exposed to lipid peroxidations. X. Zhang designed a novel DKK1 multiepitope DNA vaccine and evaluated its bone protective effects on collagen-induced arthritis (CIA), which provide a potential treatment for bone erosion in RA. S. Joplin examined the effectiveness of measures to improve patient medication adherence and proposed a new approach to patient education using musculoskeletal ultrasound. M. Westergaard studied the humoral immune response against Epstein-Barr virus (EBV) in patients with rheumatoid arthritis and found that RA patients had elevated antibodies of all isotypes characteristics of latent EBV infection, notably, for IgM and IgA (but not IgG); these were associated with the presence of characteristic RA autoantibodies.

This special issue covers many important aspects in inflammatory joint diseases, which will surely provide us with a better understanding about the pathogenesis, diagnosis, and treatment of inflammatory joint diseases.

*Guixiu Shi* *Julian L. Ambrus* *Shuang Ye* *Long Shen*

